# Glucagon-like peptide-1 stimulates acute secretion of pro-atrial natriuretic peptide from the isolated, perfused pig lung exposed to warm ischemia

**DOI:** 10.3389/frtra.2022.1082634

**Published:** 2022-12-06

**Authors:** Emilie Balk-Møller, Mathilde M. B. Hebsgaard, Nikolaj B. Lilleør, Christian H. Møller, Jens P. Gøtze, Hannelouise Kissow

**Affiliations:** ^1^ Department of Biomedical Sciences, Faculty of Health and Medical Sciences, University of Copenhagen, Copenhagen, Denmark; ^2^ Department of Cardiothoracic Surgery, Rigshospitalet, Copenhagen University Hospital, Copenhagen, Denmark; ^3^ Department of Clinical Biochemistry, Rigshospitalet, Copenhagen University Hospital, Copenhagen, Denmark

**Keywords:** *ex vivo* lung perfusion, GLP-1, ANP, pigs, warm ischemia

## Abstract

Glucagon-like peptide-1 (GLP-1) has proven to be protective in animal models of lung disease but the underlying mechanisms are unclear. Atrial natriuretic peptide (ANP) is mainly produced in the heart. As ANP possesses potent vaso- and bronchodilatory effects in pulmonary disease, we hypothesised that the protective functions of GLP-1 could involve potentiation of local ANP secretion from the lung. We examined whether the GLP-1 receptor agonist liraglutide was able to improve oxygenation in lungs exposed to 2 h of warm ischemia and if liraglutide stimulated ANP secretion from the lungs in the porcine *ex vivo* lung perfusion (EVLP) model. Pigs were given a bolus of 40 µg/kg liraglutide or saline 1 h prior to sacrifice. The lungs were then left *in vivo* for 2 h, removed *en bloc* and placed in the EVLP machinery. Lungs from the liraglutide treated group were further exposed to liraglutide in the perfusion buffer (1.125 mg). Main endpoints were oxygenation capacity, and plasma and perfusate concentrations of proANP and inflammatory markers. Lung oxygenation capacity, plasma concentrations of proANP or concentrations of inflammatory markers were not different between groups. ProANP secretion from the isolated perfused lungs were markedly higher in the liraglutide treated group (area under curve for the first 30 min in the liraglutide group: 635 ± 237 vs. 38 ± 38 pmol/L x min in the saline group) (*p* < 0.05). From these results, we concluded that liraglutide potentiated local ANP secretion from the lungs.

## Introduction

The role of atrial natriuretic peptide (ANP) was first recognized more than 40 years ago when it was shown that granules in the atrial myocytes of the heart contains a substance that lowers blood pressure (1). Today, we know that the blood pressure lowering effects of ANP involves combined effects of vasorelaxation, enhancement of microvascular permeability, natriuresis and diuresis, and antagonism of the renin-angiotensin system (2). ANP is stored in vesicles in the atrial cardiomyocytes and is secreted upon atrial stretch or hormonal activation (3). While ANP is mainly recognized for its blood pressure lowering capabilities, multiple studies have shown effects of ANP in other tissues/glands, including adipose, adrenal, brain, heart, and the lung (2, 4–8). In 1987 and 1989, two studies reported that ANP was not only secreted by the heart but also to a lesser degree from the lungs of hamsters and rats (9, 10). Furthermore, other studies have shown vaso- and bronchodilatory effects of ANP in rabbits, guinea pigs and cows (11–14). In humans, plasma ANP concentrations are increased in different disease states of the lung (15–19) and ANP has bronchodilatory effects in patients with asthma (20). Taken together, these studies suggest a role for ANP in respiratory disease.

Another hormone with multiple effects is the gut hormone glucagon-like peptide-1 (GLP-1). GLP-1 is a peptide hormone secreted from the entero-endocrine L-cells in response to nutrient intake. GLP-1 was initially recognized for its glucose-dependent stimulation of insulin secretion from the pancreatic β-cells (21). Long-acting GLP-1 receptor agonists (GLP-1RAs) were hence developed for the treatment of type 2 diabetes. More recently, GLP-1 analogues have been approved for the treatment of obesity due to its inhibitory effects of gastric emptying and food intake (22, 23). Beside its glycemic and anorectic effects, other studies have shown other functions of the hormone including inhibition of gastric acid secretion (24), protection of the intestine from acute injury (25), promotion of intestinal growth (26, 27), inhibition of bone reabsorption (28, 29) and inhibition of inflammation (30, 31). Recently GLP-1RA was found to prevent Alzheimer's disease development in mice (32).

The GLP-1 receptor (GLP-1R) is expressed in both rodent (33–35) and human lungs (36, 37) and studies have shown numerous beneficial effects of GLP-1RA in the respiratory system. These include stimulation of surfactant release from rat pneumocytes (38–40) and human lung cells (41), attenuation of lipopolysaccharide-induced lung-injury in mice (36, 42–45) as well as acute lung injury induced by H9N2 influenza virus (46) and respiratory syncytial virus (47). The mechanisms of action is not well understood, but it is suggested that GLP-1RA exerts an anti-inflammatory effects both locally in the lung (48–52) as well as systemic (30, 53).

A few studies have examined GLP-1 and pulmonary function in mice with pulmonary obstructive disease; either by measuring lung function using whole-body pletysmography or airway responsiveness to a methacholine challenge. In both settings, bronchoconstriction was alleviated after treatment with a GLP-1RA (43, 49, 54) which suggests a relaxing effect in smooth muscle cells of the bronchi. Furthermore, Balk-Møller et al. have found a dilating effect on isolated bronchi from mice were observed, which were exaggerated in bronchi from mice with obstructive disease (43).

Besides effects on the bronchi, other studies have found dilating effect on the vessels. In one, GLP-1R was localized to the vascular smooth muscles of the rat pulmonary artery and when activated, was found to induce relaxation of isolated pre-constricted pulmonary arteries (34). Another study found that GLP-1RA reduced the vascular tone in isolated perfused and ventilated rat lungs (55).

Several clinical studies have reported a decrease in blood pressure in patients receiving anti-diabetic treatment with GLP-1 analogues (56–58) and a study in mice suggested that the blood pressure lowering capabilities of GLP-1 involved GLP-1 stimulated ANP release from the heart (59). It is not known yet whether GLP-1 also stimulates ANP secretion from the lungs.

Donor organ shortage is a limiting factor in lung transplantation (60). Usually only lungs from brain dead donors are used, due to the risk of primary graft dysfunction after warm ischemia following circulatory arrest in lungs from circulatory death donors (61). The *ex vivo* lung perfusion (EVLP) technique was developed for the evaluation and reconditioning of donor lungs from brain death donors (62) however, this method also allows us to study the behavior of lungs exposed to warm ischemia in experimental settings. We recently found that pig lungs exposed to 1, but not 2, hours of warm ischemia fulfilled the transplantation criteria of PaO_2_ >13 kPa at 21% oxygen (63). This narrow time span still limits the use of lungs from circulatory death donors. In the latter experiment, we also found an increase in pulmonary vascular resistance (PVR) in pig lungs exposed to both 1 and 2 h of warm ischemia (not significant from control pigs).

Because GLP-1 has shown both anti-inflammatory effects as well as a relaxing effect on smooth muscle cells, we hypothesized that treatment with a GLP-1RA could preserve the lungs exposed to 2 h of warm ischemia. Furthermore, the use of the EVLP systems allowed us to determine whether the porcine lung secretes ANP when stimulated by a GLP-1RA.

## Material and methods

### Pigs

The study was conducted with permission from the Danish Animal Experiments Inspectorate (License No. 2015-15-0201-00573) and a local project plan was approved from the Department of Experimental Medicine (EMED), University of Copenhagen. Female Danish domestic pigs were housed at EMED according to national legislation. Experiments were performed in accordance with the Directive 2010/63/EU of the European Parliament and of the Council of 22 September 2010 on the protection of animals used for scientific purposes. All staff participating in the pig experiments had the legally compulsory courses in laboratory animal sciences. Experiments were conducted in the facility of EMED.

### Operative procedure

15 pigs (51–63 kg) were anesthetized and intubated according to general principles of EMED and heparin (20,000 IE) was administered. Catheters were placed in the jugular vein and carotid artery. Pigs were randomized to control (*n* = 6) or liraglutide groups (*n* = 9). The liraglutide group received a bolus of 40 µg/kg liraglutide (Novo Nordisk, Bagsværd, Denmark) intravenously (64, 65) 1 h before cardiac arrest. Control pigs were given saline. Liraglutide were chosen due to the fast enzymatic degradation of native GLP-1.

One hour after liraglutide/saline injection cardiac arrest by ventricular fibrillation was induced with a 9-voltage battery touching the apex of the heart through the diaphragm. 1,500 ml blood was harvested from each pig. Using a Cell Saver®5 system (Haemonetics, Massachusetts, US) washed red blood cells were extracted from the full blood. Pigs were left untouched for 2 h.

After the 2 h of warm ischemia a median sternotomy was performed, the pulmonary artery was cannulated, inferior and superior vena cavae were ligated and the ascending aorta was clamped. One liter of Custodiol® (Essential Pharmaceuticals, LLC, Durham, NC, USA) 4°C was infused through the pulmonary artery. To unload the heart the auricle of the left atrium was opened. After infusion of the pulmoplegia the heart and lungs was removed *en bloc*. The heart was carefully dissected from the lungs leaving the four pulmonary veins open.

### *Ex vivo* lung perfusion (EVLP)

**Figure 1:**
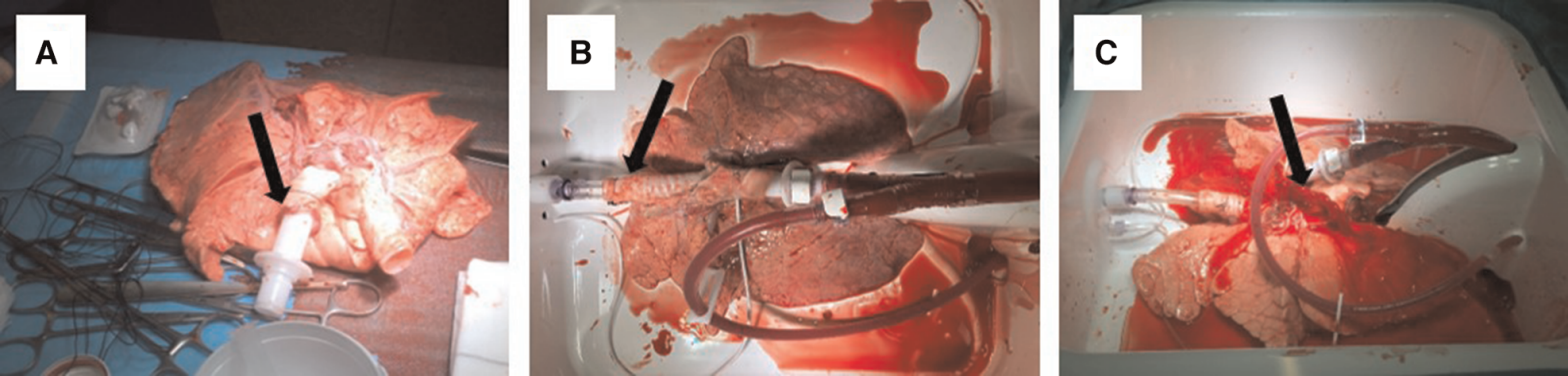
Lungs before EVLP with a tube in the pulmonary artery (arrow) (**A**), placed in the EVLP machinery with a tube in trachea (arrow) (**B**) and perfused and ventilated (**C**), notice flow from the pulmonary veins (arrow).

The lungs were connected to the Vivoline LS1 EVLP system (Vivoline Medical, Lund, Sweden) through tubes in the pulmonary artery and trachea (Figure 1A). A mechanical ventilator was connected through the tube placed in trachea (Figure 1B). Lungs were perfused in a recirculating system with Steen™ solution (XVIVO Perfusion AB, Göteborg, Sweden) mixed with autologous washed red blood cells to reach a haematocrit of 12%–14% (Figure 1C).

In the perfusion buffer of lungs from the liraglutide group a bolus of 1.125 mg liraglutide was added to reach a final concentration of 240 nM (65).

Upon connection to the EVLP, flow rate was set to 1.5 L/min and slowly raised to 4.0 L/min when reaching normothermia. The lungs were slowly warmed with perfusion buffer until reaching 37°C. At 32°C, ventilation of the lungs was started; initially, with low tidal volumes (2–3 ml/kg) and FiO_2_ 0.50, which was slowly increased to a target tidal volume of 6–8 ml/kg, a peak expiratory end pressure of 5 cm H_2_O and a respiratory frequency of 12/min. At 37°C and a PaO_2_ >40 kPa the oxygen supply was disconnected from the oxygenator and the lung was ventilated with the fraction of inspiratory oxygen (FiO_2_) of 1.00 for 10 min and evaluated before FiO_2_ was reduced to 0.21 for 10 min and lungs were evaluated again. Primary endpoints were oxygenation capacity, PVR and dynamic lung compliance.

### Sample collection

Blood samples from the pig were obtained at 0, 10 and 60 min after liraglutide/saline injection. Samples of the perfusion buffer were collected from the pulmonary vein at 0, 20, 30, 40 and 60 min after start of the perfusion. The blood and perfusion samples were immediately transferred to ice cold EDTA-containing tubes and centrifuged (1,600 × *g*, 15 min at 4°C). The supernatant was immediately placed on dry ice and stored at −80°C until further analysis. Biopsies were taken from the middle lung lobe using a stapler (Tri-StapleTM, EndoGia, Covidien) before and after EVLP. Biopsies were fixed in 4% paraformaldehyde or snap frozen and stored at −80°C until analysis.

Samples for arterial gasses were taken from the pulmonary vein during EVLP and analyzed (ABL90 Flex Radiometer A/S, Copenhagen, Denmark).

### Biochemical and histological evaluations

Liraglutide concentrations were measured in plasma and perfusion effluents by use of an in-house radioimmunoassay (66) specific for the intact N-terminus, antibody code no. 98203 (67) using liraglutide as standard.

An in-house immunoassay for porcine proANP was used to measure the total proANP concentrations in plasma and perfusion effluents (68). This method was used as ANP itself is very labile and most likely will be rapidly degraded in perfusion buffer. The assay is based on a polyclonal antibody raised against the C-terminus of proANP 1–16 measuring the C-terminal epitope after enzymatic cleavage with trypsin prior to immune measurement. This principle processing-independent analysis, thus allows detection of all possible circulating proANP fragments as previously described (68). In addition, this technology by-passes degradation issues prior to analysis, as the epitope needs to be released by *in vitro* trypsin treatment (69, 70). Assay variation was always <10% within the analytical range.

Snap frozen biopsies from before and after EVLP were analysed for myeloperoxidase (MPO) as previously described (71). In short, tissue samples were homogenised in 50 mM K- phosphate buffer pH 6.0 with 50 mM hexadecyltrimethylammonium bromide (Sigma-Aldrich, Søborg, Denmark), snap frozen on dry ice followed by thawing in water. This step was repeated three times. Samples were centrifuged (16,000 × *g*, 30 min, at room temperature) and supernatants were loaded onto a 98-well plate with 190 μl substrate buffer (50 mM K-phosphate buffer, 0.167 mg/ml O-dianisidine dihydrochloride (Sigma-Aldrich, Søborg, Denmark), and 0.0005% hydrogen peroxide (H_2_O_2_). The change in absorbance was measured at 450 nm and MPO activity were calculated using the extinction coefficient for H_2_O_2_ (1.13 × 104 M^−1^ cm^−1^) defining one unit as the amount of enzyme that degrades 1 μmol H_2_O_2_/min. Results were given as a mean of the two biopsies from each time point. For measuring IL-8 in lung tissue, biopsies were weighed, homogenised in phosphate buffered saline and assayed for porcine IL-8 (ELISA kit LS-F5330-1 LifeSpan BioSciences, Seattle, WA, USA) following manufacturer's instructions.

Fixed tissue was embedded in paraffin and sections of 4 µm were stained with hematoxylin and eosin and scored according to Haam 2015 (72). Alveolar congestion, haemorrhage, leukocyte infiltration and thickness of the alveolar wall were scored with a score of 0–4 (0 = normal lung, 1 ≤ 25%, 2 = 25%–50%, 3 = 50%–75%, 4 ≥ 75% lung involvement). Scores was summed to reach a final histopathological score. The origin of the sample were blinded to the observer.

### Statistics

Data are expressed as mean with standard error of the mean (SEM). EVLP data (PaO_2_, PaCO_2_, PVR and compliance at FiO_2_ 1.00 and 0.21), proANP concentrations in plasma and accumulated proANP concentrations in perfusion samples were compared using an unpaired Students *T* test with Welch's correction. MPO activity, IL8 concentrations and histopathological score were compared using a one way Analysis of Variance (ANOVA) followed by Šídák's multiple comparisons test.

Dose-time relations of proANP concentrations and difference between the two groups were tested by a two-way ANOVA. Area under curve for proANP secretion in perfusion samples were analysed using an unpaired Students *T* test with Welch's correction.

In all cases, *p* < 0.05 was considered significant. Tests were performed in GraphPad Prism 7.0 (La Jolla, USA).

## Results

### Oxygenation capacity, PVR and dynamic lung compliance

**Figure 2:**
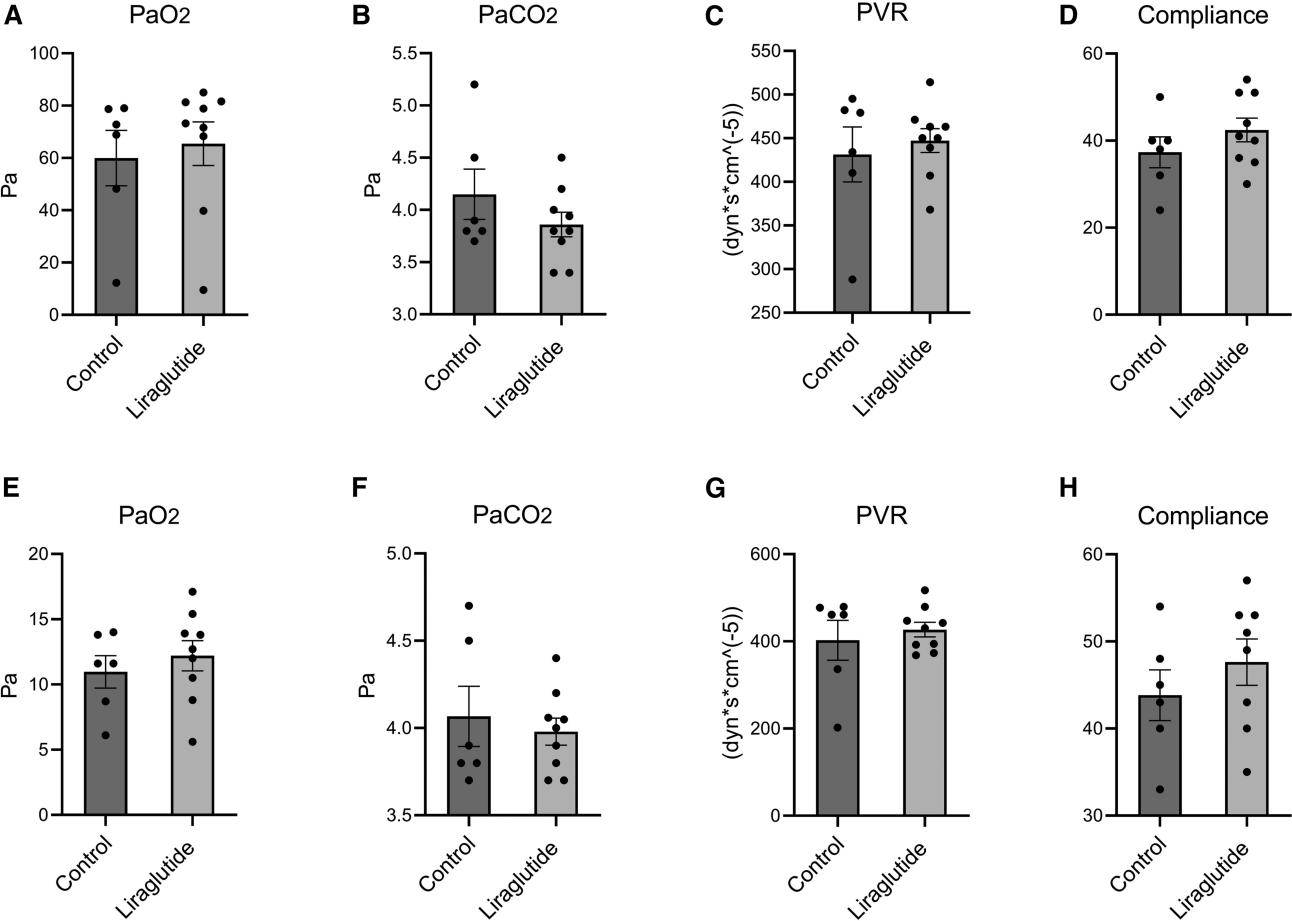
Oxygenation capacity, pulmonary vascular resistance (PVR) and dynamic lung compliance in lungs after 10 min at FiO_2_ 1.00 (**A–D**) and 10 min at FiO_2_ 0.21 (**E–H**). Data is shown as mean ± SEM, *n* (saline) = 6, *n* (liraglutide) = 9. Statistics were performed using an unpaired Students *T* test with Welch's correction.

PaO_2_ at FiO_2_ 1.00 were 65.5 ± 8.3 kPa in the liraglutide group and 60.0 ± 10.6 kPa in the control group, this was not statistically different (Figure 2A). Likewise, the oxygenation capacity at FiO_2_ 0.21 was not different between the two groups with 12.20 ± 1.2 kPa and 11.0 ± 1.3 in the liraglutide and control group respectively (Figure 2E). PCO_2_, PVR and compliance were similar in the two groups at both FiO_2_ 1.00 and FiO_2_ 0.21 (Figures 2B–D,F–H).

### Liraglutide and proANP concentrations

**Figure 3:**
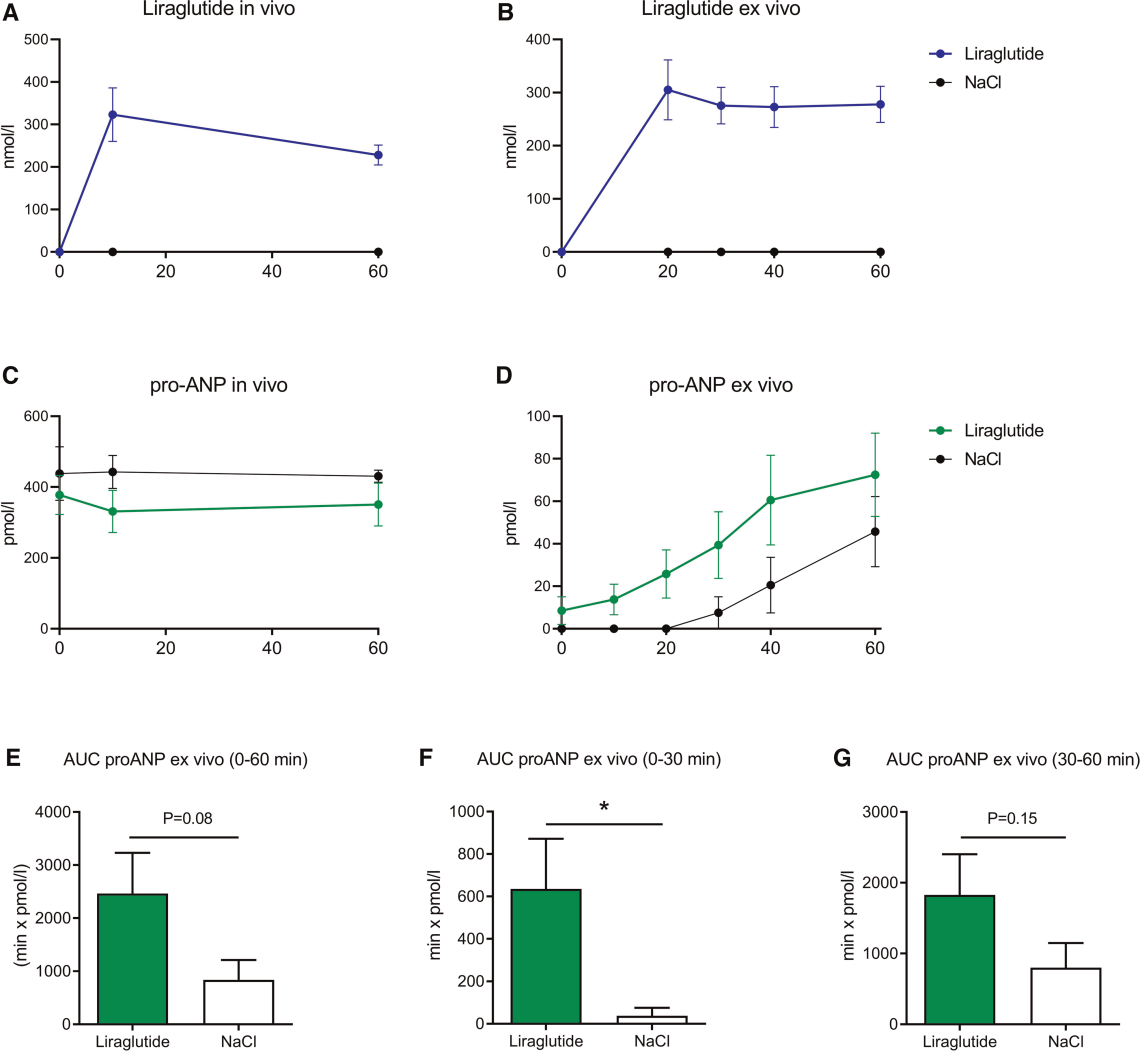
Plasma concentrations of liraglutide (**A**) and proANP (**C**) over time after a bolus of 40 µg/kg liraglutide or saline in pigs. Concentration of liraglutide (**B**) and proANP (**D**) over time in perfusion buffer added 1.125 mg liraglutide or saline. AUC of proANP in perfusion buffer for 0–60 min (**E**), first 30 min (**F**) and last 30 min (**G**). Data is shown as mean ± SEM, *n* (saline) = 6, *n* (liraglutide) = 9. *p < 0.05. Statistics were performed using a two-way ANOVA.

Liraglutide showed stable levels throughout the entire experimental period, both during the *in vivo* period and the *ex vivo* phase. Mean plasma concentrations were 323 ± 20 and 228 ± 24 nmol/L at 20 and 60 min after injection, respectively. *Ex vivo*, the concentration of liraglutide was approximately 300 nmol/L (Figures 3A,B). *In vivo*, there was no difference in proANP concentrations in plasma between the two groups (Figure 3C). However, the *ex vivo* concentrations in buffer showed a marked increase during the first phase of the *ex vivo* time. ProANP concentrations increased over time in both groups but significantly more in the liraglutide group (*p* < 0.01) (Figure 3D). Area under curve (AUC) of proANP was 2,464 ± 768 pmol/L x min in the liraglutide groups vs. 839 ± 909 pmol/L x min in the saline group (Figure 3E). AUC values for the first 30 min were almost 17-fold larger in the liraglutide group; 635 ± 237 vs. 38 ± 38 pmol/L x min (*p* < 0.05) (Figure 3F). AUC values for 60 min was doubled in the liraglutide group, however this was not statistically different between groups (Figure 3G).

### Inflammatory markers

**Figure 4:**
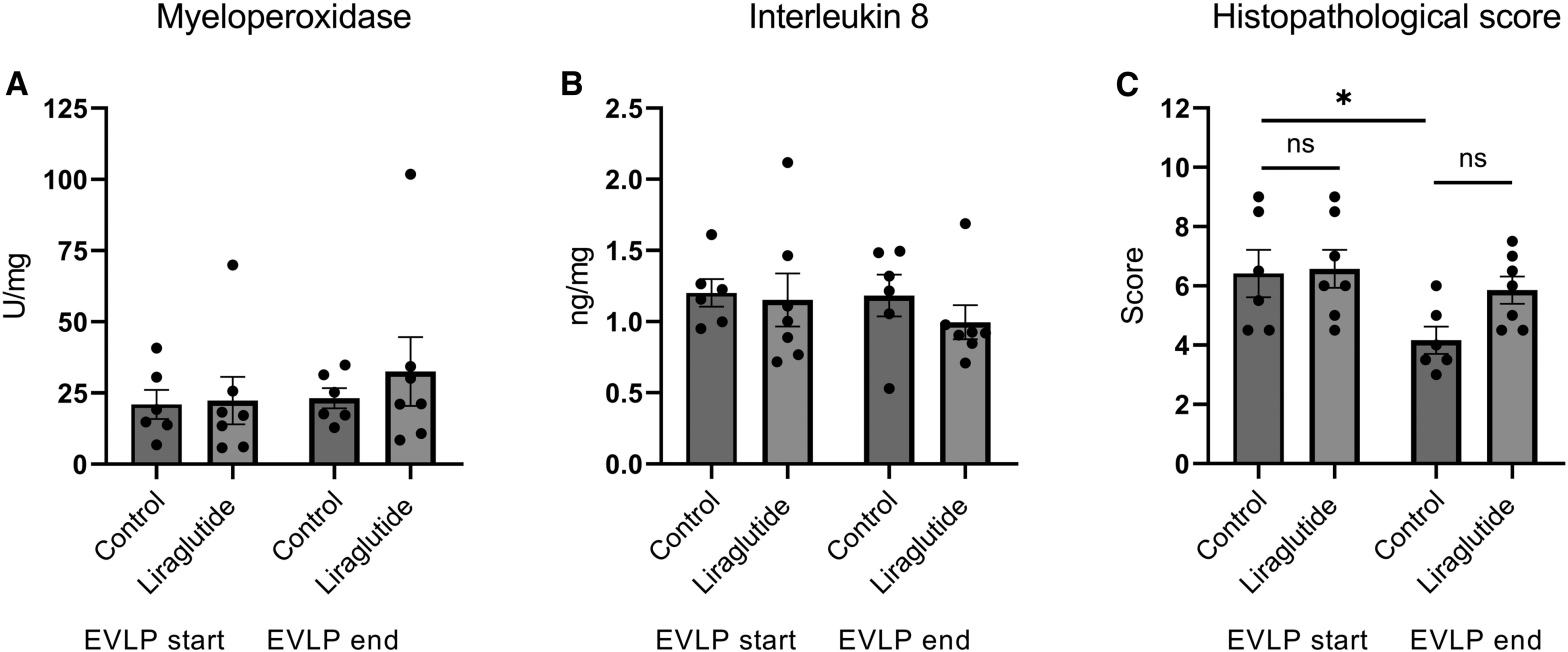
Activity of MPO (**A**), concentrations of IL8 (**B**) and histopathological score (**C**) in lung biopsies obtained in the start and in the end of EVLP. *p < 0.05. Statistics were performed using a one way Analysis of Variance (ANOVA) followed by Šídák's multiple comparisons test.

We measured the activity of myeloperoxidase and the content of interleukin 8 in biopsies taken when EVLP started and at the end of EVLP. We found no differences in these markers (Figures 4A,B). The histopathological score decreased during EVLP, however this was only significant in the control group (Figure 4C). It was mainly the scores of alveolar congestion, that decreased during EVLP.

## Discussion

ANP and GLP-1/GLP-1RA have both been shown to have protective effects in the lungs; GLP-1 in animal models of lung disease (73–75) and ANP in humans with asthma (20, 76). While the protective effects of ANP have been suggested to be mediated through direct bronchodilatory effects (20), the mechanisms underlying the protective effects of GLP-1 remain elusive.

In this study we investigated if treatment with a GLP-1RA were able to rescue lungs exposed to 2 h of warm ischemia. However, we found no improvement in oxygenation capacity when animals were treated with liraglutide 1 h before sacrifice.

The acceptance threshold for human transplantation is 13 kPa at FiO_2_ 0.21 (77) which two out of four in the control group and four out of 9 in the liraglutide group reached. These finding are different from our previous study in which all lungs exposed to 2 h of warm ischemia failed to reach the threshold (63). We have no good explanation of this, besides the increased hands-on experience of the staff. We changed the perfusion buffer from a homemade solution to using the original Steens solution™, but the effect of this is unknown. Another discrepancy to our former study is the inter group variations. As an example PO_2_ at FiO_2_ 0.21 in the 2 h ischemia grouped had a range of 9.8–11.9 kPa in our previous study (63), while there was a range from 6.1 to 14.0 kPa in the control group in this study. This is a major limitation since beneficial effect of GLP-1 could be masked due to the low power of the study. We also had severe outliers in each group with a very low oxygenation capacity. We suspect that this very low capacity could be due to bad and variable health of domestestic pigs. As this was not confirmed with tests we did not exclude them from the study.

PVR, PaCO_2_, and lung compliance of all lungs were acceptable, with not differences between the two groups. Therefore, the hypothesized relaxing effects of liraglutide on the vascular smooth muscle cells could not be confirmed in this setup.

We did not find any differences in the analysis of IL-8 concentration, MPO activity and leucocyte infiltration from start to end EVLP, or between the liraglutide and saline treated. This could be due to the short observation period of EVLP, which does not reveal long-term effects of acute ischaemic injury. In studies with 24 h EVLP and transplantation of donor lungs, a rise of IL-8 concentration, proportional to the duration of warm ischaemia, was observed (78, 79). In the majority of studies investigating the anti-inflammatory effects of GPL-1 in the lungs, treatment was given more than 6 h before sacrifice (43, 45, 54, 80, 81). However, in a study by Yanay et al. the GLP-1RA, exendin-4 showed anti-inflammatory effect on lipopolyssacharide induced endotoxemia in rats after only 3 h. The treatment decreased IL-1α, IL-1β, IL-6, TNFα and IFNγ but not IL-10, showing GLP-1RA to have an acute effect as well. We ended the EVLP period after the second evaluation phase with FiO_2_ 0.21 to observe our primary endpoints [oxygenation capacity, pulmonary vascular resistance (PVR) and dynamic lung compliance] which means the lungs were only exposed to liraglutide for approximately 90 min. We cannot exclude that a longer exposure time of GLP-1 could have an impact on inflammation due to warm ischemia.

The histological examination of the lungs showed an improvement in the histopathological score during EVLP. This was expected since studies have shown that EVLP improves lung damage ascended from the warm ischemia (82).

As mentioned in the introduction numerous experimental studies have shown a positive effect of GLP-1RA on pulmonary diseases, including COPD, however very few clinical trials have been made, despite the fact that T2D have been treated with GLP-1RA for years, and COPD commonly co-occur with T2D (83). This provides an opportunity for shared therapies and the GLP-1R could potentially be a target in treating patients with multimorbidity. A recent RCT was investigating the effect of liraglutide (3.0 mg) in patients with obesity and COPD for 40 weeks and found increased forced vital capacity (FVC) and carbon monoxide diffusion capacity but no changes in FEV1 or FEV1/FVC. The study suffered from low power, since only 20 patients were included in each group and besides the effect on pulmonary function, the liraglutide treated also had a massive weight loss. A newer RCT included a larger patient group (76 patients) with T2D and COPD, but with a shorter treatment period with liraglutide (1.8 mg) to minimize weight loss as a potential confounding factor. They found increased FVC in the group treated with liraglutide, but no changes in FEV1 or FEV1/FVC as in the former study. Further clinical trials are warranted to elucidate the role of GLP-1RA in pulmonary diseases.

As GLP-1R expression is high in the lung (75) and because ANP was demonstrated to be secreted from rat lungs (9) and responsible for the GLP-1 mediated vasodilation seen in hypertensive mice (84) we speculated that ANP might be secreted from the pig lungs in response to stimulation with GLP-1.

We chose to measure total proANP rather than the processed and bioactive ANP, since ANP is rapidly eliminated *in vivo* and futhermore is chemically unstable *in vitro* (68). ANP is produced intracellularly by posttranslational processing of proANP. The proANP fragments are co-secreted with ANP after processing and have a longer half-life *in vivo* (85) therefore, proANP is a valid surrogate measure of ANP secretion (86–88), much like C-peptide is a valid measure of insulin secretion (89).

Our data shows that liraglutide directly stimulate proANP secretion from the lung. Liraglutide both decreased the time frame before significant increase in proANP were detected and increased the overall secretion. To our knowledge, this is the first study to show secretion of proANP from the porcine lung in general. Also, it is the first study to demonstrate that GLP-1R activation in the lung directly stimulates ANP secretion.

Our study does however, not reveal the mechanisms underlying GLP-1 stimulated ANP secretion and what physiological effects this secretion may have. We speculate that increased ANP secretion may be a compensatory mechanism to protect the lung under disease states. Although this is not a study of porcine lung disease, the perfused lungs had been deprived of oxygen for 2 h prior to placement in the EVLP machinery. The deprivation of oxygen inevitably causes tissue damage and cytokine release, therefore the secretion of ANP might be a compensatory response to the warm ischemia. The implication of this for the interpretation of our results is difficult to assess, but proANP secretion was clearly potentiated by liraglutide.

The translatability of our finding to humans may be questioned as two different human studies did not find an effect of GLP-1 on plasma ANP concentrations (90, 91). In a mouse study by Kim et al. the authors found that stimulation of GLP-1R led to secretion of ANP and lowering of blood pressure (59). The effect was abolished in GLP-1 receptor (GLP-1R) knockout mice indicating that ANP release was dependent on activation of the GLP-1R. These results prompted a follow-up study to investigate if these findings translated into man. Over a period of 21 days they treated 17 middle-aged obese, hypertensive men with liraglutide and measured ANP in plasma samples. They were unable to find increases in ANP levels as seen in the mouse study (90). Two other studies investigating both healthy young men (92) and middle-age obese men (91) came to the same conclusion. In line with these findings, we did not observe any differences in proANP secretion between treatment groups *in vivo*. Lastly, one human study did find increased plasma concentrations of ANP (and BNP) and lowering of blood pressure upon long term liraglutide treatment in patients with type II diabetes (93). The cause for these contradictory results warrants further investigation and could be due to use of different assays for ANP. It could also be speculated that the GLP-1/GLP-1RA mediated secretion from the lungs may escape detection in the circulation because the absolute GLP-1/GLP-1RA mediated ANP secretion from the lung is relatively small compared to the secretion from heart and because ANP is rapidly eliminated in the circulation and unstable even *in vitro* as mentioned above. This concept is well-known for other hormones and perhaps best illustrated by the gut and pancreatic hormone somatostatin, which both inhibits the secretion of gut hormones and glucagon and insulin (94–97), although plasma concentrations only increase minimally or not at all (98).

However, even if that is not the case, our results may still have physiological significance as ANP secretion from the lung may act by paracrine mechanisms. We did not see any effect on oxidative capacity or PVR of GLP-1/ANP axis in our perfused lung, but we cannot rule out that the relative short perfusion time mask an effect. At any rate, more studies are needed to conclude whether the GLP-1R mediated ANP secretion in the perfused lung can be translated to any beneficial vasodilatory or bronchodilatory effect, improving the resuscitation of human donor lungs exposed to warm ischemia in the EVLP system.

In conclusion, our study showed that the GLP-1RA liraglutide stimulates ANP release in a porcine perfused lung model and this is, to the best of our knowledge the first study to describe this phenomenon. A full understanding of the physiologic aspect of this mechanism requires further investigation.

## Data Availability

The raw data supporting the conclusions of this article will be made available by the authors, without undue reservation.
